# Magnetoencephalography-derived oscillatory microstate patterns across lifespan: the Cambridge centre for ageing and neuroscience cohort

**DOI:** 10.1093/braincomms/fcae150

**Published:** 2024-04-29

**Authors:** Yujing Huang, Chenglong Cao, Shenyi Dai, Hu Deng, Li Su, Ju-Sheng Zheng

**Affiliations:** Zhejiang Key Laboratory of Multi-Omics in Infection and Immunity, Center for Infectious Disease Research, School of Medicine, Westlake University, Hangzhou 310024, Zhejiang Province, China; Research Center for Industries of the Future, School of Life Sciences, Westlake University, Hangzhou 310024, Zhejiang Province, China; Westlake Laboratory of Life Sciences and Biomedicine, Hangzhou 310024, Zhejiang Province, China; Institute of Biology, Westlake Institute for Advanced Study, Hangzhou 310024, Zhejiang Province, China; Department of Neurosurgery, The First Affiliated Hospital of University of Science and Technology of China, Hefei 230001, Anhui, China; Department of Economics and Management, China Jiliang University, Hangzhou 310024, Zhejiang Province, China; Hangzhou iNeuro Technology Co., LTD, Hangzhou 310024, Zhejiang Province, China; Peking University Huilongguan Clinical Medical School, Beijing Huilongguan Hospital, Beijing 100096, China; Department of Psychiatry, University of Cambridge, Cambridge CB20SZ, United Kingdom; Neuroscience Institute, University of Sheffield, Sheffield, South Yorkshire S102HQ, United Kingdom; Zhejiang Key Laboratory of Multi-Omics in Infection and Immunity, Center for Infectious Disease Research, School of Medicine, Westlake University, Hangzhou 310024, Zhejiang Province, China; Research Center for Industries of the Future, School of Life Sciences, Westlake University, Hangzhou 310024, Zhejiang Province, China; Westlake Laboratory of Life Sciences and Biomedicine, Hangzhou 310024, Zhejiang Province, China; Institute of Biology, Westlake Institute for Advanced Study, Hangzhou 310024, Zhejiang Province, China

**Keywords:** machine learning, microstates, healthy aging, alpha

## Abstract

The aging brain represents the primary risk factor for many neurodegenerative disorders. Whole-brain oscillations may contribute novel early biomarkers of aging. Here, we investigated the dynamic oscillatory neural activities across lifespan (from 18 to 88 years) using resting Magnetoencephalography (MEG) in a large cohort of 624 individuals. Our aim was to examine the patterns of oscillation microstates during the aging process. By using a machine-learning algorithm, we identify four typical clusters of microstate patterns across different age groups and different frequency bands: left-to-right topographic MS1, right-to-left topographic MS2, anterior-posterior MS3 and fronto-central MS4. We observed a decreased alpha duration and an increased alpha occurrence for sensory-related microstate patterns (MS1 & MS2). Accordingly, theta and beta changes from MS1 & MS2 may be related to motor decline that increased with age. Furthermore, voluntary ‘top-down’ saliency/attention networks may be reflected by the increased MS3 & MS4 alpha occurrence and complementary beta activities. The findings of this study advance our knowledge of how the aging brain shows dysfunctions in neural state transitions. By leveraging the identified microstate patterns, this study provides new insights into predicting healthy aging and the potential neuropsychiatric cognitive decline.

## Teaser

Yujing Huang *et al.* investigated the dynamic oscillatory neural activities across lifespan. It was found that there were dominant age-related alpha band microstate effects, especially an increased alpha occurrence in the whole brain with healthy aging. A higher-order sensory and posterior network may be recruited with the increase of age.

## Graphical Abstract

**Graphical abstract:**
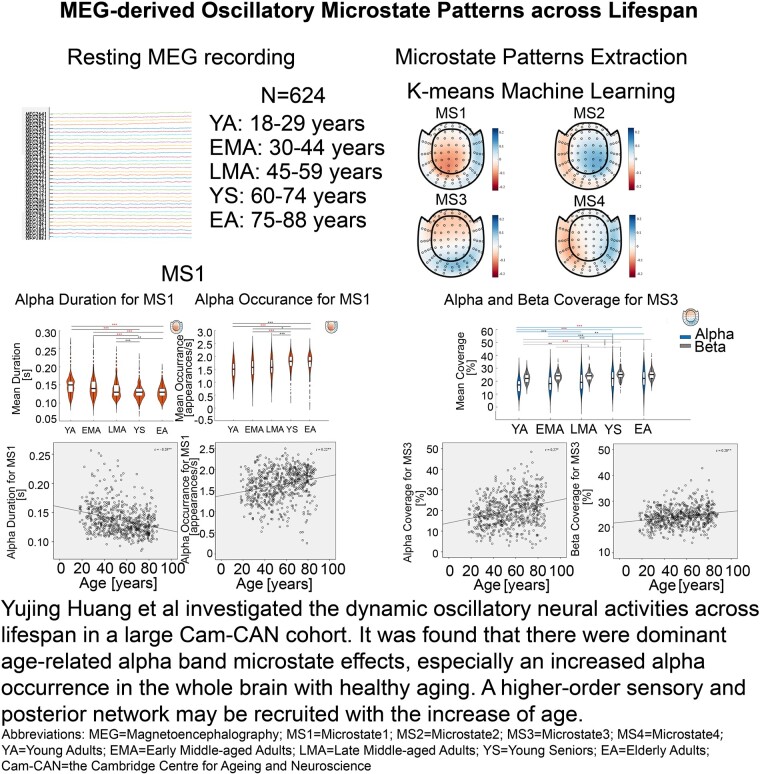

## Introduction

The neural dynamic oscillatory patterns represent an early hallmark of aging.^1,2^ It has been posited that monitoring regular and predictable oscillations over the course of an adult’ lifespan can aid in identifying potential progression of cognitive decline.^3,4^ At the cellular level, neurons have bio-electrochemical properties that facilitate the flow of electrical ions, resulting in the production of electromagnetic fields.^5^ There are five typical oscillatory brain signals in humans: delta, theta, alpha, beta and gamma. Abnormal neural physiological activities within specific frequency bands can serve as potential histopathological biomarkers for brain dysfunctions.^6-8^ So far, the specific contribution of oscillatory changes within certain frequency bands on healthy aging are not yet well understood.

There are substantial changes in alpha oscillation during aging in humans, such as alpha slowing,^9^ alpha power reduction,^10,11^ alpha reactivity declining,^12,13^ and alpha sub-component changes.^14^ Additionally, other oscillatory neurons and topology reflect different activities with the increasing age. For instance, Barry and De Blasio^15^ observed reduced theta power and increased beta power, accompanied by decreased alpha power in elder adults. Furthermore, the neuropsychological Stroop task, a standard attention conflict measurement, highlights the opposing alpha and theta activities.^16^ Thus, there is no consensus regarding whether aging involves multi-frequency dynamic oscillatory changes or is characterized by dominant alpha frequency deficiency. Previous Cam-CAN studies^17-20^ have indicated reduced neural efficiency or specificity rather than compensation across lifespan. For example, Tibon *et al.*^17,18^ showed that there was an age-related ‘neural shift’ with decreased occurrence of ‘lower-order’ networks in early visual states and increased occurrence of ‘higher-order’ fronto-temporal-parietal networks in visual and sensorimotor states. Another leading theory,^19,20^ the posterior-to-anterior shift in aging (PASA), states that the anterior regions are recruited when posterior cortical function is impaired.

Koenig *et al*.^21^ defined four classes: A (left-to-right orientation), B (right-to-left orientation), C (anterior-posterior orientation) and D (fronto-central maxium). Brain sources underlying microstates in the literature showed that different microstates were highly correlated with functional magnetic imaging neural activities: auditory network (microstate A), visual network (microstate B), saliency network (microstate C) and attention network (microstate D).^22,23^ Magnetoencephalography (MEG) is a non-invasive measurement of oscillatory magnetic fields with excellent temporal resolution and reasonable spatial resolution. In the present study, using resting MEG, we aimed to record the spontaneous rhythmic responses across lifespan and perform machine learning-based microstate clustering in a cohort study involving participants from different age groups. We hypothesized that alpha rhythm changes would be the most pronounced MEG microstate phenomenon in the aging brain. We further hypothesized that the microstates originating from the posterior regions would move anteriorly with age to increase the occurrence of higher-order sensory systems.

## Materials and methods

### Demographics

The present study was based on the cohort of Cambridge Centre for Ageing and Neuroscience (Cam-CAN), involving 624 participants ranging from 18–88 years. The demographic details in the study are shown in Table 1. Participants were divided into five groups: young adults (YA, 18–29 years old), early middle-aged adults (EMA, 30–44 years old), late middle-aged adults (LMA, 45–59 years old), young seniors (YS, 60–74 years old) and elderly adults (EA, 75–88 years old). We performed resting MEG session for all these participants. The study was approved by the Cambridgeshire 2 Research Ethics Committee and all participants provided written informed consent prior to the study.

**Table 1:** Summary of participant demographics

Group	N	Age (years)	Mean Age (mean ± S.D.)	Gender (female/male)	Handedness (R/L)
Young Adults	67	18–29	25.16 ± 3.42	38/29	61/6
Early Middle-aged Adults	141	30–44	37.27 ± 4.07	68/73	126/14^a^
Late Middle-aged Adults	150	45–59	51.73 ± 4.30	73/77	139/11
Young Seniors	150	60–74	66.86 ± 4.10	75/75	135/14^a^
Elderly Adults	116	75–88	79.88 ± 3.23	56/60	111/5
Total	624	18–88	54.48 ± 18.26	310/314	572/50

^a^The handedness record of one participant was ambidexter.

### Resting MEG recordings

MEG data were recorded via 306-channel VectorView MEG system (Elekta Neuromag, Helsinki). MEG Vectorview system contains 204 planar gradiometers and 102 magnetometers. Magnetometers consist of a single coil to measure the magnetic flux perpendicular to the cortex surface. Planar gradiometers are arranged in pairs (a ‘figure-of-eight’ coil configuration) and the differences between two loops of the spatial gradient were calculated. The signals from planar gradiometers indicate magnetic fields from two directions in a plain parallel to the head surface. Participants were required to keep eyes closed but stay awake during resting MEG recording in a magnetically shielded room. MEG resting-state data were recorded with a duration of 8 and 40 sec, sampled at 1 kHz with a high-pass filter of 0.03 Hz. Head positions within MEG helmet were estimated via Head-Position Indicator (HPI) coils for offline correction of head movements.

### Resting MEG data preprocess

The MaxFilter 2.2.12 software (Elekta Neuromag Oy, Helsinki, Finland) applied temporal Single Source Separation (‘t-SSS’) to preprocess continuous MEG data.^24,25^ T-SSS helps to detect and reconstruct noisy MEG channels, remove noises from external sources, correct head-motion artefacts and remove 50 Hz noise. Following the de-noising steps, continuous MEG data were imported into MATLAB and resampled into 250 Hz via SPM12 (http://www.fil.ion.ucl.ac.uk/spm). Then MEG data were filtered and segmented into different frequency bands: delta (1–3 Hz), theta (4–7 Hz), alpha (8–13 Hz), beta (14–30 Hz), gamma (31–40 Hz) and overall (1–40 Hz). We applied two-pass butter-worth filters, initially a high-pass filtre and then a low-pass filter with zero phase shift. Specifically, for delta, theta, alpha, beta, gamma and overall frequency bands, the high-pass filters were 1, 4, 8, 14, 31 and 1 Hz respectively; the low-pass filters were 3, 7, 13, 30, 40 and 40 Hz respectively. The filtered data for each frequency band were continuous datasets across experimental time. After that, we firstly combined original planar channels (i.e. MEGPLANAR electrodes) into MEGCOMB electrodes by using SPM12. Then original magnetometers (i.e. MEGMAG electrodes) and newly MEGCOMB electrodes were concatenated for better data interpretation in further microstates analyses. However, to compare the global explained variance for MEGCOMB electrodes with MEGMAG electrodes, we would re-run the preprocesses and separately conduct microstate global explained variance analyses for MEGCOMB electrodes or MEGMAG electrodes (Table 2).

**Table 2:** Global explained variance for gradiometer only and magnetometer only electrodes

GEV(%)	Com (Mean ± S.E.)	MAG (Mean ± S.E.)	Paired-sample *T*-tests (t_df_)
Overall	78.32 ± 0.07	35.63 ± 0.16	t_623_ = 331.19***
Delta	78.87 ± 0.07	37.00 ± 0.22	t_623_ = 210.83***
Theta	78.08 ± 0.07	34.21 ± 0.13	t_623_ = 332.43***
Alpha	78.77 ± 0.10	40.33 ± 0.31	t_623_ = 157.98***
Beta	78.27 ± 0.06	33.57 ± 0.12	t_623_ = 463.10***
Gamma	76.67 ± 0.08	25.72 ± 0.09	t_623_ = 444.62***

‘***’ indicated that *P* < 0.001; all age groups were included for each frequency band. COM showed gradiometer electrodes while MAG was magnetometer electrodes.

### Microstate pattern analyses using machine learning

For each oscillatory frequency band, the modification of the microstate k-means algorithm was used to extract microstate patterns.^26,27^ We applied + microstate (MATLAB package, https://github.com/lukewtait/microstate_toolbox) which had high signal-to-noise ratio for spontaneous transitions between brain states. The MEG microstate pipeline was based on machine-learning k-means pipeline presented by Pascual-Marqui *et al*.^28^ Details and justification of the microstate pipeline are illustrated as follows.

1. Import resting MEG sensor-level preprocessed data. The clean MEG dataset included 204 channels in total, consisting of 102 magnetometer electrodes and 102 combined gradiometer electrodes.
2. Extract activity maps at global-field power (GFP) peaks per individual. The GFP at a time point was defined as the standard deviation of the magnetic potential across sensors. Subsequent clustering required samples with optimal signal-to-noise and topographic stability, which correspond to those with peaks in the GFP. Thereby, polarity of the map was ignored;
3. Run k-means clustering on maps per individual. K maps were chosen as the initial cluster centroids (*k* = 4). Then we calculated cosine distance between activity maps and centroids. Each activity map was subsequently clustered based on nearest centroid. Using the new centroids, the procedure of calculating cluster labels and updating cluster centroids was iterated until convergence was reached. Due to random initial seeding, the k-means algorithm was repeated 20 times and the repetition with highest global explained variance (GEV) was chosen for further analyses;^21^
4. Run two-level ‘global clustering’ to obtain global map per age group per frequency band, and cluster the global microstate maps from each individual at the group level;
5. Backfit group-level global maps to obtain individual microstate parameters (duration, coverage, occurrent) per age group per frequency band. Specific parameters include duration (mean duration of a specific microstate that remains stable), coverage (the percentage of time spent when a given microstate is dominant), occurrence (the number of times the microstate appears per second) *et al*.
6. Re-order global maps per age group per frequency band, so as to obtain comparable microstate parameters across individuals at different age group and different frequency bands. The rule of re-ordering was based on Pearson’s correlations of global maps between young group at overall frequency band (1–40 Hz) and other groups. Clustering analyses in + microstate toolbox had variations in topology across different age groups or across different frequency bands. The aim of global map correlations is to re-order the clustering maps to obtain microstates with consistent topology. In this case, we would fit-back the comparable statistics after re-ordering for further analyses.
7. Calculate microstate statistics per microstate pattern per age group per frequency band. The parameters of microstates (coverage, occurrence, duration) were compared via one-way analysis of variance (ANOVA) across different age group per frequency band. The *post hoc* tests were analyzed via the Tukey method if there was significant homogeneity of variances; Tamhane’s T2 was used for multiple comparisons if there was significant heterogeneity of variances.
8. Correlation analyses between age and microstate statistics. We analyzed Pearson’s correlations between age and microstate parameters (coverage, occurrence, duration) respectively per frequency band.
9. Visualizing microstate data via neuromag306 template.
10. Global Explained Variance (GEV) comparison for gradiometer and magnetometer electrodes. For each frequency band, the GEV was computed for ‘gradiometer only’ and ‘magnetometer only’ respectively. Then paired-sample *T*-tests were used to compare the GEV differences for gradiometer and magnetometer electrodes per frequency band regardless of age groups.

### Statistical analysis

Chi-square tests were conducted for age differences per age group and handedness differences per age group. The demographic differences across age groups were analyzed by Chi-square tests. One-way ANOVA was used to compare the age-related group differences per frequency band per microstate pattern per microstate parameter (coverage, occurrence and duration). The *post hoc* tests were analyzed via the Tukey method if there was significant homogeneity of variances; Tamhane’s T2 was used for multiple comparisons if there was significant heterogeneity of variances. The Pearson’s correlations between age and each microstate parameter were analyzed per microstate pattern per frequency band. The Bonferroni method was used for multiple statistical correction of one-way ANOVA, *post hoc* tests and correlations. Multiple linear regression was analysed with age as a dependent variable and all other parameters as independent variables. Microstate GEV was compared via pair-sample *T*-tests between MEGCOMB and MEGMAG electrodes. The group-level correlations among microstate maps were based on the Pearson’s correlations.

## Results

### Demographics

According to Chi-square tests, there were no statistical differences in gender distributions per age group for the recruited participants [YA: χ^2^(1) = 1.20, *P* = 0.27; EMA: χ^2^(1) = 0.17, *P* = 0.67; LMA: χ^2^(1) = 0.10, *P* = 0.74; YS: χ^2^(1) = 0, *P* = 1; EA: χ^2^(1) = 0.13, *P* = 0.71]. Additionally, there was no significant gender difference across age group [χ^2^(4) = 1.60, *P* = 0.80]. Furthermore, the statistic results about handedness showed that there were fewer left-hand participants than right-hand participants per age group [YA: χ^2^(1) = 45.14, *P* < 0.001; EMA: χ^2^(1) = 89.6, *P* < 0.001; LMA: χ^2^(1) = 109.22, *P* < 0.001; YS: χ^2^(1) = 98.26, *P* < 0.001; EA: χ^2^(1) = 96.86, *P* < 0.001]. Similarly, there was no significant handedness difference across age group [χ^2^(4) = 3.45, *P* = 0.48].

### Determination of microstate patterns

**Figure 1:**
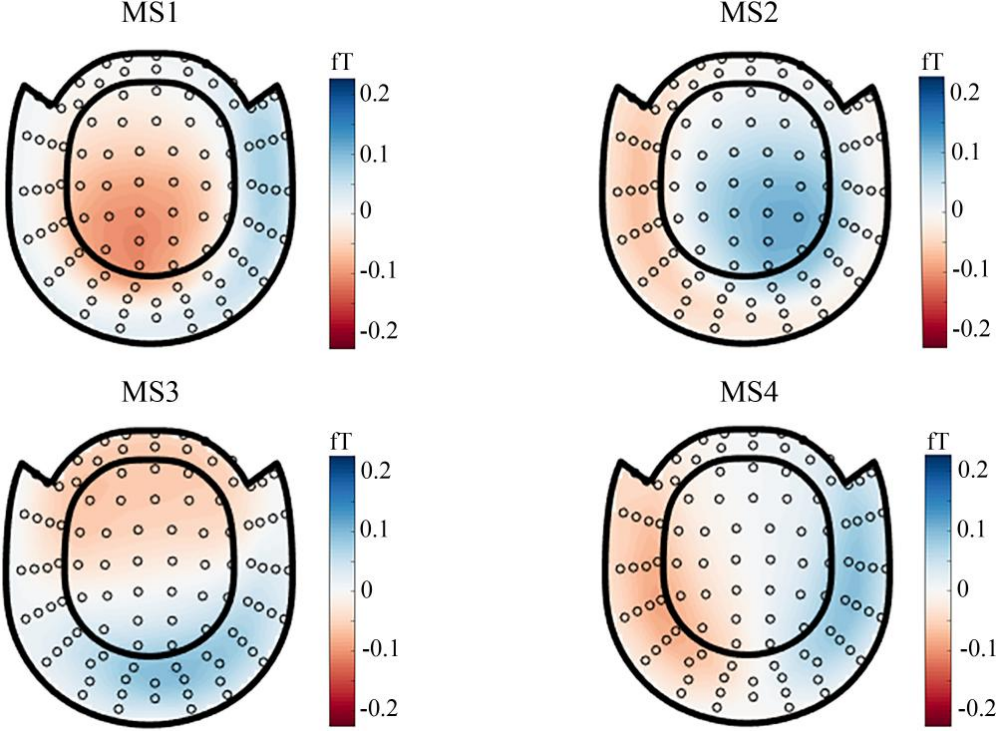
**Determination of microstate patterns.** MS1 and MS2 exhibited a left-to-right orientation and a right-to-left orientation respectively. MS3 indicated an anterior-posterior orientation while a fronto-central maximum was shown by MS4. The polarity was marked via blue and red: blue indicated positive and red was negative. The microstate patterns are group-level maps of young adults at 1–40 Hz. ‘MS1’, ‘MS2’, ‘MS3’ and ‘MS4’ indicate ‘microstate1’,’microstate2’,’microstate3’ and ‘microstate4’ respectively in the figure. The unit of microstate global map is ‘fT’.

**Figure 2:**
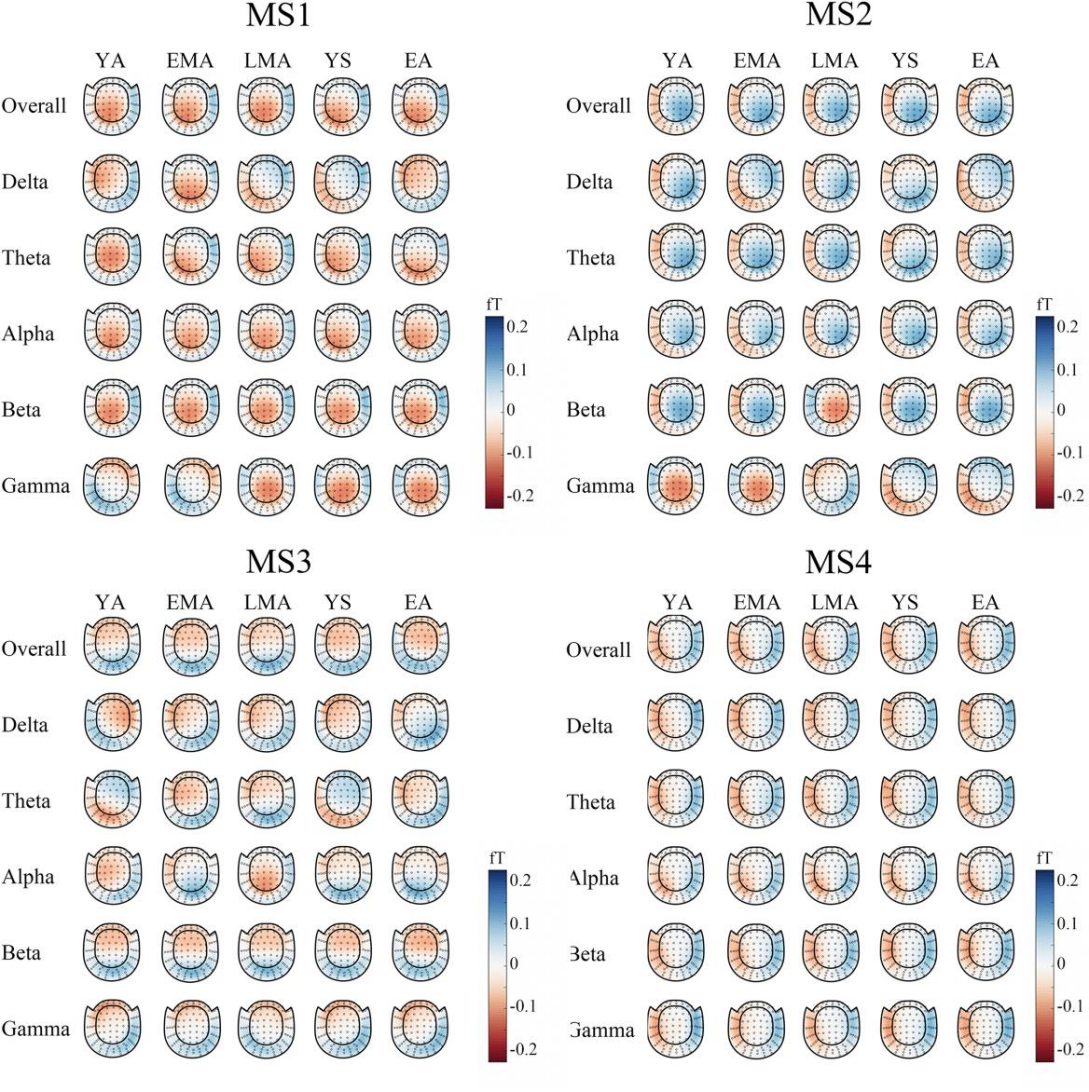
**Microstate patterns across age groups and frequency bands.** Four typical clusters (MS1, MS2, MS3 and MS4) were illustrated. Each column showed an age group and each row indicated a frequency band (Overall:1–40 Hz; Delta: 1–3 Hz; Theta:4–7 Hz; Alpha:8–13 Hz; Beta: 14–30 Hz; Gamma: 31–40 Hz). The blue and red for polarity can be ignored as suggested by previous researchers [32–34]. Five age groups were defined: young adults (YA, 18–29 years old), early middle-aged adults (EMA, 30–44 years old), late middle-aged adults (LMA, 45–59 years old), young seniors (YS, 60–74 years old) and elderly adults (EA, 75–88 years old). ‘MS1’, ‘MS2’, ‘MS3’ and ‘MS4’ indicate ‘microstate1’,’microstate2’,’microstate3’ and ‘microstate4’ respectively in the figure. The unit of microstate global map is ‘fT’.

**Figure 3:**
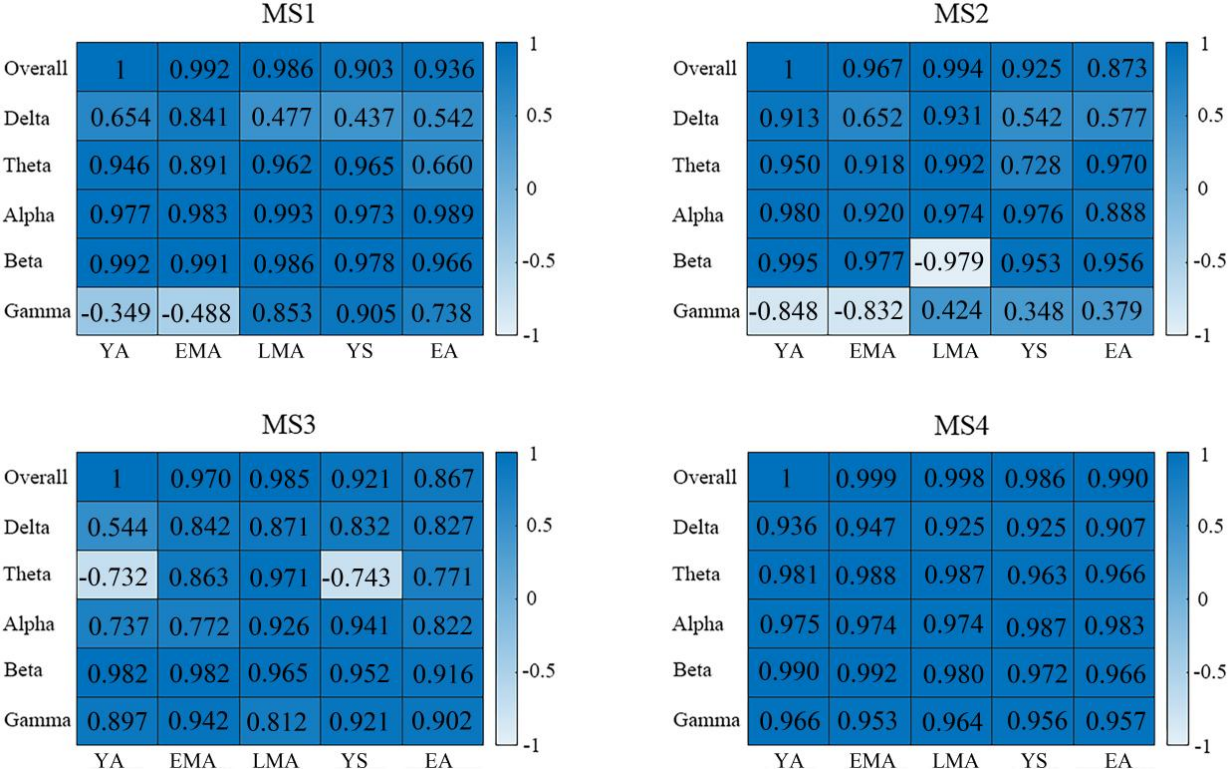
**The global map correlation results.** The Pearson’s correlations between young group at overall frequency (1–40 Hz) and other groups were illustrated per microstate pattern at group level. Each column indicated an age group while each row showed a frequency band. All correlations were significant (*r*-values for Pearson’s correlation analyses, *P* ≤ 0.01). Five age groups were defined: young adults (YA, 18–29 years old), early middle-aged adults (EMA, 30–44 years old), late middle-aged adults (LMA, 45–59 years old), young seniors (YS, 60–74 years old) and elderly adults (EA, 75–88 years old). Each row represents a frequency band (Overall:1–40 Hz; Delta: 1–3 Hz; Theta:4–7 Hz; Alpha:8–13 Hz; Beta: 14–30 Hz; Gamma: 31–40 Hz). Correlation coefficients range from −1.0 to +1.0 with unit free. ‘MS1’, ‘MS2’, ‘MS3’ and ‘MS4’ indicate ‘microstate1’,’microstate2’,’microstate3’ and ‘microstate4’ respectively in the figure.

The observed topographic clusters were initially labeled by Koenig *et al*. (1999) as class A, B, C and D.^21,22^ Microstate map A (MS1) and map B (MS2) exhibited a left-to-right orientation and a right-to-left orientation respectively. Microstate map C (MS3) indicated an anterior-posterior orientation while a fronto-central maximum was shown by microstate map D (MS4) (Fig. 1). As shown in Fig. 2, these four maps seem to consistently dominate the resting MEG data across different age groups and different frequency bands. As suggested by previous studies,^23,29,30^ the polarity can be ignored in the microstate definition (blue versus. red in Fig. 1 & Fig. 2). For each microstate pattern, the correlations between the young group at overall frequency band (1–40 Hz) and other groups were calculated. The correlation results (mostly *r* > 0.90, Fig. 3) help to re-order the microstates patterns for different age groups and different frequency bands. Thus, the parameters of microstates across age group and oscillatory activities were comparable for a given microstate pattern after re-ordering.

### Higher gradiometer global explained variance for dynamic microstate patterns

As reported in previous studies, the optimal number of clusters by using the machine-learning model (i.e. K-means clustering) is four microstate clusters. The GEV of four cluster maps explained varies among studies, ranging from 65% to 84%.^19^ Our findings indicated that gradiometer electrodes showed higher GEV compared to magnetometer electrodes for all frequency bands (Table 2). These observations might represent that the set of four cluster microstate patterns fit the common map for gradiometers rather than magnetometers.

### Alpha duration decrease and occurrence increase for sensory networks

**Figure 4:**
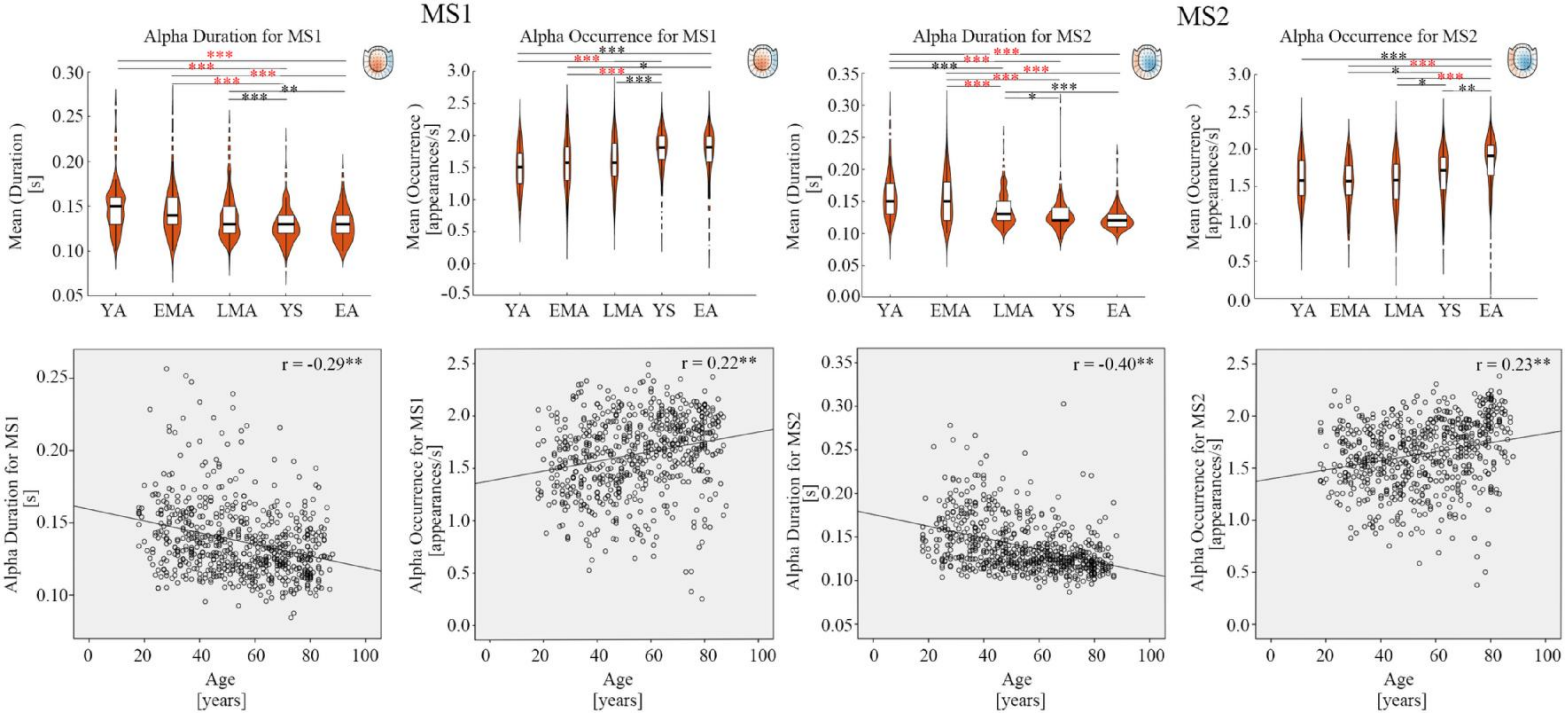
**Alpha duration decrease and occurrence increase for MS1&MS2.** The violin plots had age groups as X axis and mean value of microstate parameters (duration or occurrence) as Y axis. The black symbols ‘*’，‘**’, ‘***’ indicated the *post hoc* uncorrected significance level of ‘*P* ≤ 0.05’, ‘*P* ≤ 0.01’ and ‘*P* ≤ 0.001’ respectively. The red symbol of ‘***’ indicated that the *P*-value survived after Bonferroni correction. The *post hoc* tests for MS1 alpha occurrence and MS2 alpha occurrence were analyzed via the Tukey method since there was significant homogeneity of variances; the *post hoc* tests for MS1 alpha duration and MS2 alpha duration via the Tamhane’s T2 since there was significant heterogeneity of variances. Five age groups were defined: young adults (YA, 18–29 years old), early middle-aged adults (EMA, 30–44 years old), late middle-aged adults (LMA, 45–59 years old), young seniors (YS, 60–74 years old) and elderly adults (EA, 75–88 years old). The units in the violin plots for duration and occurrence were ‘second’ and ‘times’ respectively. The bottom scatter plots were the correlations results between age and microstate parameters (duration or occurrence). Each data point represents the mean value per participant. The X axis of scatter plots were age and Y axis of scatter plots were mean value of microstate parameters (duration or occurrence). Correlation coefficients range from −1.0 to +1.0 with unit free. The uncorrected ‘*P* < 0.01’ values of Pearson’s correlations were marked with the symbol ‘**’. The Bonferroni correction for Pearson’s correlations was used but no significant correlations survived. The units in the scatter plots for age, duration and occurrence were ‘years’, ‘second’ and ‘times’ respectively. ‘MS1’ and ‘MS2’ indicate ‘microstate1’ and ‘microstate2’ respectively in the figure.

As mentioned above, MS1 and MS2 were indications of auditory and visual networks respectively. In our findings, there was significant decrease in alpha duration and increase in alpha occurrence for MS1 and MS2 (Fig. 4). In other words, aging adults showed decreased alpha duration time of stable MS1 or MS2 while there were increased number of times per second for the appearance of MS1 or MS2 for alpha activities [Alpha Duration: *F*(4619)_MS1_ = 15.06, *P*_MS1_ < 0.001, *F*(4619)_MS2_ = 32.57, *P*_MS2_ < 0.001; Alpha Occurrence: *F*(4619)_MS1_ = 10.14, *P*_MS1_ < 0.001, *F*(4619)_MS2_ = 13.55, *P*_MS2_ < 0.001]. The microstate patterns of alpha were significantly correlated with age [Alpha Duration: *R*^2^_MS1_ = 0.08, *r*_MS1_ = −0.29, *P*_MS1_ < 0.01, *R*^2^_MS2_ = 0.16, *r*_MS2_ = −0.40, *P*_MS2_ < 0.01; Alpha Occurrence: *R*^2^_MS1_ = 0.05, *r*_MS1_ = 0.22, *P*_MS1_ < 0.01, *R*^2^_MS2_ = 0.05, *r*_MS2_ = 0.24, *P*_MS2_ < 0.01].

**Figure 5:**
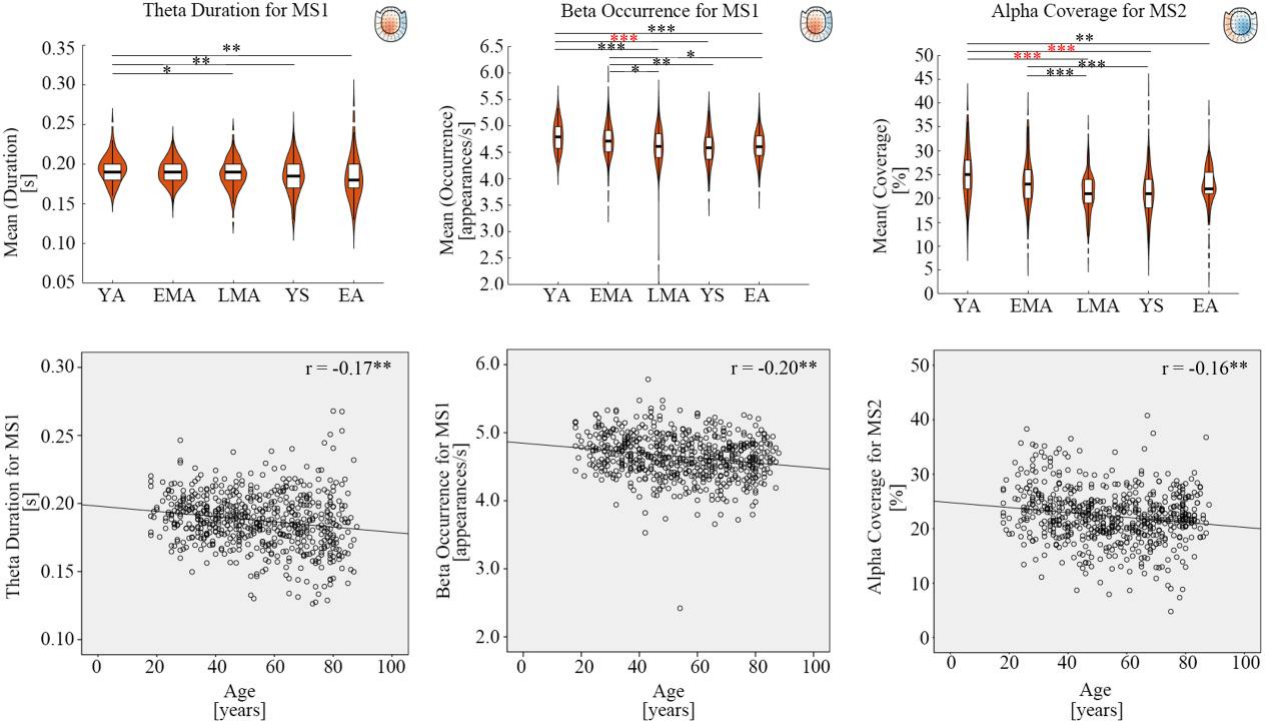
**Multi-frequency changes for MS1&MS2 across lifespan.** Apart from dominant alpha oscillation decline across lifespan, there was accompanying decreased theta duration and decreased beta occurrence for MS1. Also, decreased alpha coverage was observed for MS2. The violin plots had age groups as X axis and mean value of microstate parameters (duration, occurrence or coverage) as Y axis. The black symbols ‘*’，’**’, ‘***’ indicated the post-hoc uncorrected significance level of ‘*P* ≤ 0.05’, ‘*P* ≤ 0.01’ and ‘*P* ≤ 0.001’ respectively. The red symbol of ‘***’ indicated that the *P*-value survived after Bonferroni correction. The *post hoc* tests for MS1 beta occurrence and MS2 alpha coverage were analyzed via the Tukey method since there was significant homogeneity of variances; the *post hoc* tests for MS1 theta duration via the Tamhane’s T2 since there was significant heterogeneity of variances. Five age groups were defined: young adults (YA, 18–29 years old), early middle-aged adults (EMA, 30–44 years old), late middle-aged adults (LMA, 45–59 years old), young seniors (YS, 60–74 years old) and elderly adults (EA, 75–88 years old). The units in the violin plots for duration, occurrence and coverage were ‘second’, ‘times’ and ‘percentage’ respectively. The bottom scatter plots were the correlations results between age and microstate parameters (duration, occurrence or coverage). Each data point represents the mean value per participant. The X axis of scatter plots were age and Y axis of scatter plots were mean value of microstate parameters (duration, occurrence or coverage). Correlation coefficients range from −1.0 to +1.0 with unit free. The uncorrected ‘*P* < 0.01’ values of Pearson’s correlations were marked with the symbol ‘**’. The Bonferroni correction for Pearson’s correlations was used but no significant correlations survived. The units in the scatter plots for age, duration, occurrence and coverage were ‘years’, ‘second’, ‘times’ and ‘percentage’ respectively. ‘MS1’ and ‘MS2’ indicate ‘microstate1’ and ‘microstate2’ respectively in the figure.

Furthermore, other frequency bands, such as theta band or beta band, showed accompanying decline with age for MS1 (Fig. 5). For example, the ANOVA and correlation results showed that theta duration and beta occurrence for MS1 were decreased across lifespan [Theta duration: *F*(4619)_MS1_ = 4.33, *P*_MS1_ = 0.002, *R*^2^_MS1_ = 0.03, *r*_MS1_ = −0.17, *P*_MS1_ < 0.01; Beta occurrence: *F*(4619)_MS1_ = 8.35, *P*_MS1_< 0.001, *R*^2^_MS1_ = 0.04, *r*_MS1_ = −0.20, *P*_MS1_ < 0.01]. Similarly, we observed decreased alpha coverage for MS2 across lifespan [Alpha coverage: *F*(4619)_MS2_ = 11.87, *P*_MS2_ < 0.001, *R*^2^_MS2_ = 0.03, *r*_MS2_ = −0.17, *P*_MS2_ < 0.01]. *post hoc* analyses of MS1 & MS2 were summarized in Table 3 (details in Supplementary Table 1).

**Table 3:** Microstate pattern parameter changes with healthy aging

MS1	YA N = 67	EMA N = 141	LMA N = 150	YS N = 150	EA N = 116	One-way ANOVA F (df1 = 4, df2 = 619)	*post hoc* Tukey	Pearson’s r (2-tailed)^b^	Beta
Alpha/duration	0.14(3E3)	0.14(2E3)	0.14(2E3)	0.12(1E3)	0.12(1E3)	F = 15.06***	YA = EMA>>>YS = EA(a)	−0.29**	n.s.
Alpha/occurrence	1.47(3E2)	1.56(3E2)	1.58(3E2)	1.75(2E2)	1.69(3E2)	F = 10.14***	YA = EMA<<<YS	0.22**	n.s.
Beta/occurrence	4.79(3E2)	4.72(2E2)	4.61(2E2)	4.58(2E2)	4.60(2E2)	F = 8.35***	YA>>>YS	−0.20**	n.s.
Gamma/occurrence	6.70(8E2)	6.69(4E2)	6.18(6E2)	5.98(6E2)	5.94(8E2)	F = 25.13***	YA = EMA>>>LMA = YS = EA(a)	−0.34**	n.s.

MS2	YA N = 67	EMA N = 141	LMA N = 150	YS N = 150	EA N = 116	One-way ANOVA F (df1 = 4, df2 = 619)	*Post hoc* Tukey	Pearson’s r (2-tailed)^b^	Beta
Delta/occurrence	0.53(6E3)	0.56(4E3)	0.52(6E3)	0.47(1E2)	0.55(9E3)	F = 24.15***	YA>>>YS EMA>>>LMA EMA>>>YS YS<<<EA(a)	−0.12**	n.s.
Alpha/coverage	0.25(6E3)	0.23(4E3)	0.21(3E3)	0.21(4E3)	0.22(4E3)	F = 11.87***	YA>>>LMA = YS	−0.16**	−0.51**
Alpha/duration	0.15(4E3)	0.15(2E3)	0.13(2E3)	0.12(1E3)	0.12(1E3)	F = 32.57***	EMA>>>LMA YA = EMA>>>YS = EA(a)	−0.40**	n.s.
Alpha/occurrence	1.60(3E2)	1.55(2E2)	1.56(2E2)	1.66(2E2)	1.80(3E2)	F = 13.55***	EMA<<<EA LMA<<<EA	0.23**	0.64***
Gamma/coverage	0.22(4E3)	0.22(2E3)	0.25(2E3)	0.24(3E3)	0.25(5E3)	F = 14.59***	YA = EMA<<<LMA(a)	0.22**	n/a
Gamma/occurrence	6.00(7E2)	6.08(4E2)	6.82(5E2)	6.61(5E2)	6.66(5E2)	F = 42.93***	YA = EMA<<<LMA = YS = EA	0.34**	−0.55*

MS3	YA N = 67	EMA N = 141	LMA N = 150	YS N = 150	EA N = 116	One-way ANOVA F (df1 = 4, df2 = 619)	*Post hoc* Tukey	Pearson’s r (2-tailed)^b^	Beta
Delta/coverage	0.21(3E3)	0.23(3E3)	0.22(3E3)	0.19(6E3)	0.19(6E3)	F = 14.12***	EMA>>>YA EMA>>>EA EMA>>>YS(a)	−0.20**	0.85**
Delta/occurrence	0.53(5E3)	0.57(4E3)	0.55(5E3)	0.47(6E3)	0.48(1E2)	F = 32.07***	YS<<<YA<<<EMA EMA>>>YS = EA LMA>>>YS = EA(a)	−0.32**	−0.55**
Theta/occurrence	1.17(1E2)	1.20(1E2)	1.20(1E2)	1.15(1E2)	1.25(1E2)	F = 10.01***	YA = YS<<<EA(a)	0.07*	n.s.
Alpha/coverage	0.16(6E3)	0.18(5E3)	0.19(6E3)	0.21(6E3)	0.21(7E3)	F = 10.74***	YA<<<YS = EA(a)	0.27**	n.s.
Alpha/occurrence	1.23(4E2)	1.36(3E2)	1.45(3E2)	1.64(3E2)	1.68(4E2)	F = 17.75***	YA = EMA<<<YS = EA	0.33**	n.s.
Beta/coverage	0.22(4E3)	0.23(2E3)	0.24(3E3)	0.24(3E3)	0.24(3E3)	F = 7.45***	YA<<<YS	0.20**	n.s.
Beta/duration	0.05(4E4)	0.05(3E4)	0.05(3E4)	0.05(3E4)	0.05(3E4)	F = 14.43***	YA<<<LMA = YS = EA	0.27**	n.s.

MS4	YA N = 67	EMA N = 141	LMA N = 150	YS N = 150	EA N = 116	One-way ANOVA F (df1 = 4, df2 = 619)	*Post hoc* Tukey	Pearson’s r (2-tailed)^b^	Beta
Alpha/occurrence	1.76(3E2)	1.84(2E2)	1.86(2E2)	1.99(2E2)	1.95(3E2)	F = 10.43***	YA<<<YS	0.23**	n.s.

Notes:The symbols of ***, >>>, <<< indicate that significance level are corrected based on Bonferroni method. The values of ‘mean(S.E.)’ was provided for each age group (YA: young adults; EMA: early middle age; LMA: late middle age; YS: young seniors; EA: elder adults). The letter ‘(a)’ means Tamhane’s T2 was used due to heterogeneity of variances. Pearson’s correlations between age and microstates parameters were calculated at the uncorrected significance level of 0.05 (‘*’) and 0.01 (‘**’). The superscript letter ‘b’means that the statistics didn’t pass the Bonferroni correction. Beta of linear regression analyses indicated the standardized coefficients with age as dependent variable and all microstate parameters as independent variables.

### Increased alpha occurrence but reverse Beta changes for salience/attention networks

**Figure 6:**
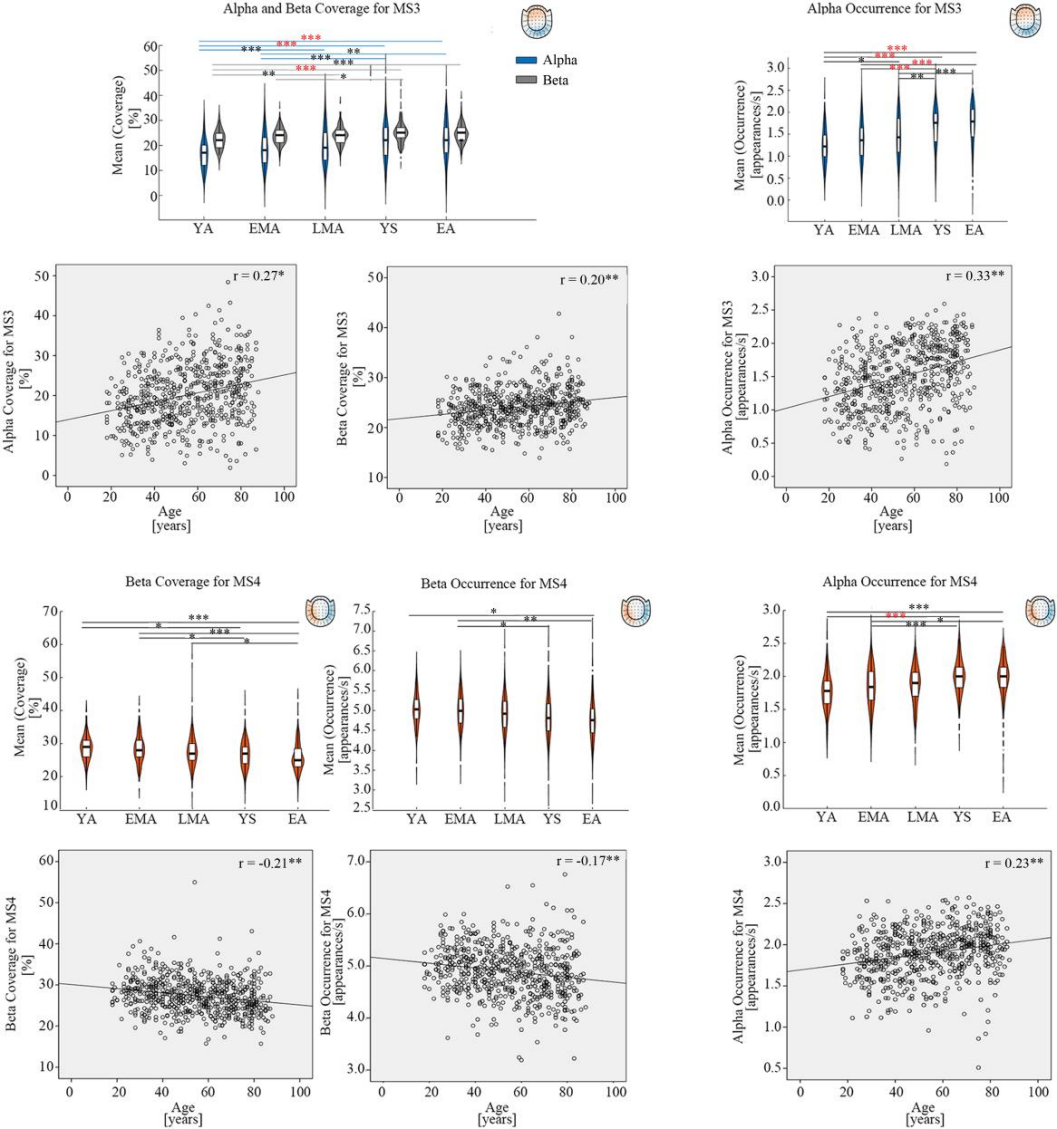
**Alpha and Beta changes for MS3&MS4 across lifespan.** The violin plots had age groups as X axis and mean value of microstate parameters (occurrence or coverage) as Y axis. The black symbols ‘*’，’**’, ‘***’ indicated the *post hoc* uncorrected significance level of ‘*P* ≤ 0.05’, ‘*P* ≤ 0.01’ and ‘*P* ≤ 0.001’ respectively. The red symbol of ‘***’ indicated that the *P*-value survived after Bonferroni correction. The *post hoc* tests for MS3 beta coverage, MS3 alpha occurrence, MS4 alpha occurrence, MS4 beta coverage and MS4 beta occurrence were analyzed via the Tukey method since there was significant homogeneity of variances; the *post hoc* tests for MS3 alpha coverage via the Tamhane’s T2 since there was significant heterogeneity of variances. Five age groups were defined: young adults (YA, 18–29 years old), early middle-aged adults (EMA, 30–44 years old), late middle-aged adults (LMA, 45–59 years old), young seniors (YS, 60–74 years old) and elderly adults (EA, 75–88 years old). The units in the violin plots for occurrence and coverage were ‘times’ and ‘percentage’ respectively. The bottom scatter plots were the correlations results between age and microstate parameters (occurrence or coverage). Each data point represents the mean value per participant. The X axis of scatter plots were age and Y axis of scatter plots were mean value of microstate parameters (occurrence or coverage). Correlation coefficients range from −1.0 to +1.0 with unit free. The uncorrected ‘*P* < 0.01’ values of Pearson’s correlations were marked with the symbol ‘**’. The Bonferroni correction for Pearson’s correlations was used but no significant correlations survived. The units in the scatter plots for age, occurrence and coverage were ‘years’, ‘times’ and ‘percentage’ respectively. ‘MS3’ and ‘MS4’ indicate ‘microstate3’ and ‘microstate4’ respectively in the figure.

MS3 and MS4 may reflect salience and attention networks.^18,19^ As shown in Fig. 6, we found that MS3 and MS4 had increased alpha occurrence, which was consistent with the findings for MS1 and MS2. [Alpha occurrence: *F*(4619)_MS3_ = 17.75, *P*_MS3_ < 0.001, *R*^2^_MS3_ = 0.11, *r*_MS3_ = 0.33, *P*_MS3_ < 0.01; *F*(4619)_MS4_ = 10.44, *P*_MS4_ < 0.001, *R*^2^_MS4_ = 0.05, *r*_MS4_ = 0.26, *P*_MS4_ < 0.01]. The results indicated that there was aging-related increased number of times per second for whole-brain microstate patterns of alpha responses.

The possible aging-related compensatory changes of MS3 and MS4 were shown for beta band (Fig. 6). Interestingly, alpha and beta coverage was increased for MS3 while three beta parameters (coverage, occurrence, duration) were declined for MS4 [Alpha coverage: *F*(4619)_MS3_ = 10.75, *P*_MS3_ < 0.001, *R*^2^_MS3_ = 0.07, *r*_MS3_ = 0.28, *P*_MS3_ < 0.01; Beta coverage: *F*(4619)_MS3_ = 7.45, *P*_MS3_< 0.001, *R*^2^_MS3_ = 0.04, *r*_MS3_ = 0.20, p_MS3_ < 0.01; *F*(4619)_MS4_= 7.12, *P*_MS4_ < 0.001, *R*^2^_MS4_ = 0.05, *r*_MS4_ = −0.23, *P*_MS4_< 0.01; Beta occurrence: *F*(4619)_MS4_ = 4.89, *P*_MS4_ = 0.001, *R*^2^_MS4_ = 0.03, *r*_MS4_ = −0.20, *P*_MS4_ < 0.01; Beta duration: *F*(4619)_MS4_ = 5.01, *P*_MS4_ = 0.001, *R*^2^_MS4_ = 0.03, *r*_MS4_ = −0.18, *P*_MS4_ < 0.01]. Coverage was defined as the percentage of time spent when a given microstate was dominant. Thus, MS3 had an increased average percentage of time but overall MS4 patterns were declineed especially for beta band across lifespan. *Post hoc* analyses of MS3 & MS4 were summarized in Table 3 (details in Supplementary Table 1).

Above all, there was dominant alpha deficiency with multi-rhythm decay across lifespan. We observed a whole-brain microstate patterns changes across lifespan. Microstate patterns for rhythmic activities showed a dominant alpha deficiency for most microstate patterns. Meanwhile, some other frequency bands had accompanying responses, such as theta and beta microstates patterns. These findings were briefly summarized in Table 4.

**Table 4:** Brief sum of aging-related oscillation microstate patterns

Microstate	Coverage	Duration	Occurrence
MS1		α↓θ↓	α↑β↓
MS2	α↓	α↓	α↑
MS3	α↑β↑		α↑
MS4	β↓	β↓	α↑β↓

## Discussion

In the present study, typical topographic clusters of microstates dominate the resting MEG data across different age groups and different frequency bands. The alpha deficiencies were apparent with increasing age, such as the whole-brain alpha occurrence abnormality. Apart from alpha occurrence changes, the decline of sensory and motor networks might be reflected via distributed oscillation microstate patterns. Specifically, we observed decreased alpha duration and increased alpha occurrence (left-to-right topographic MS1 & right-to-left topographic MS2) from young to elderly adults. Furthermore, theta duration and beta occurrence decreased across lifespan, which may be related to motor impairments. In addition, the ‘top-down’ voluntary network may be reflected by anterior-posterior MS3 and fronto-central MS4. We found that the decreased beta was apparent for MS4 during aging while there may be complementary relationships between MS3 and MS4.

### Machine-learning-identified microstate patterns with intra-subject stability

Traditionally oscillation analyses to extract power or peak frequency are meaningful, but the promising machine-learning-identified oscillatory microstates patterns may be superior to help observe the multi-frequency topology patterns with high temporal resolution. The concept of brain states is that a discrete microstate pattern remains stable before transitioning to a different state.^31,32^ Machine-learning microstate patterns rely on topologic clustering to label a couple of discrete clusters which can explain the majority of global variance across the cortex.^26,27^ Machine-learning k-means clustering was first proposed to calculate the global field power and to extract parameters (such as occurrence, duration, coverage) for each microstate pattern.^28^ Microstate analysis involves clustering the sensor-space spatial topographies without an arbitrary priori selected time window for continuous neural data. Recently, the dysfunction of brain dynamics is reflected by microstates in neurological diseases.^33-36^ Our identified microstate patterns provide insights into transitions of spontaneous brain states across lifespan.

Although the k-means++ algorithm can be used for selection of initial cluster,^37^ four cluster maps are retained in the majority of previous studies.^38-40^ It was argued that four clusters in most previous studies exhibited highly similar topographies, strongly resembling the maps initially described by Koenig and colleagues.^21^ The oscillatory brain responses observed in microstates offer a valuable perspective for gaining a deeper understanding of aging. In consistent with previous studies,^21-23^ we identified four clusters that were extracted across lifespan for different neural oscillations.

As for our findings on group-level correlations, the network activation patterns across lifespan describe intra-subject stability. The neural activities mainly include left-to-right, right-to-left, anterior-to-posterior and fronto-central topologies with healthy aging. In previous literature, four or five microstates revealed a set of brain regions active in the majority of networks.^41^ The GEV, a measure of how well the spatial microstate topographies can explain the variance of the data, can reach up to 84%.^23^ Regardless of the age group or frequency bands in our study, the common areas may correspond to the main hubs about structural/functional brain networks (e.g. anterior and posterior cingulate cortices, dorsal superior prefrontal cortex, supramarginal gyrus, insula, precuneus, superior frontal cortex *et al*.).^42,43^

The patterns related to MS4 (i.e. fronto-central topology) are highly correlated across groups and frequencies. The MS4 has been attributed to the attentional network in the fMRI literature via source localization approach.^22^ Britz *et al*. demonstrated that MS4 correlated with negative BOLD activation in right-lateralized dorsal and ventral areas of the frontal and parietal cortices. Additionally, the posterior cingulate cortices were active in all the microstates maps.^42,43^ Thus, we could probably see the important function of attention related to the stable fronto-central network across lifespan.

### Microstate patterns with intra-subject variability

It is also interesting to find that there is still intra-subject variability in the maps between groups within a frequency or in the maps between frequencies within an age group. This may indicate the individualized microstate patterns and they won’t be the exact map across age groups or across frequency bands. The MS1&MS2 variability appears at delta band and gamma band, indicating that the sensory impairments with the increase of age may be the coherence of the low frequency and high-frequency band. Previously, ketamine-induced ‘delta-to-gamma’ shift has been observed in animal studies, indicating the underlying N-methyl-D-aspartic acid (NMDA) mechanism.^44-46^ Previous researchers claimed that EEG delta-band phase and gamma-band amplitudes predict some complementary aspects of the time course of spikes of visual cortical neurons.^47^ Another possible explanation by Cam-CAN studies claimed that higher-order visual system may be more involved than lower-order visual system with healthy aging.^17,18^ These researches provide possible explanations for the delta and gamma intra-subject variability in our findings. Our findings may be consistent with previous Cam-CAN studies, indicating the recruitment of higher-order sensory system across lifespan.

The relative low group-level correlations for MS3 at theta and alpha bands may be related to the dysfunction in saliency network across lifespan. Existing literatures have showed that the salience network serves to identify salient stimuli and switch between the central executive network and the default-mode network.^48^ Older adults had lower theta power in resting electroencephalograms and in task performances.^49^ Former study also suggested that alpha-band oscillations play an important role in distractor filtering.^50^ Our current study supports the evidence that the theta/alpha may be a sensitive marker of cognitive aging in salience network.

### Sensory microstate pattern effects across lifespan

In the current study, MS1 and MS2 indicated the dysfunction in visual and auditory networks, which probably reflect the alpha changes across lifespan. We found that the microstate patterns with decreased alpha duration and increased alpha occurrence are highly valuable in advancing our understanding of healthy aging. Most researchers favor the view that there is a strong relationship between levels of sensory and cognitive decline across lifespan.^51,52^ Age-related hearing loss and visual impairments starts to develop gradually in middle adulthood (around 35–45 years) and tends to accumulate over time.^53^ Furthermore, it has been demonstrated that older adults experience a decline in grey matter within the posterior temporal areas and parietal-occipital regions at an annual rate of 2%.^54^ In addition, the precuneus, lateral parietal and temporal association cortices, and the posterior consistently had grey matter atrophy, hypometabolism and amyloid plaque deposition in normal older adults.^55-59^ The alpha changes of MS1 and MS2 may be linked to the neuropsychiatric abnormality and neural dysfunctions, involving higher-order sensory systems.

In our current study, the microstates in divergent coupling (i.e. delta, theta, gamma) oscillations for MS1 and MS2 may be related to motor dysfunction across lifespan. Prior studies have suggested that motor network dysfunctions are related to distributed oscillatory systems in the brain.^60-63^ It has been demonstrated that motor cortical theta oscillation decline emerges in the medial frontal cortex with increased age. The co-activation of central-parietal regions and frontal-central areas is supported by alternating theta oscillatory patterns in both visual and auditory modalities.^64-67^ In addition to the involvement of alpha and beta oscillations in movement generation,^68-71^ oscillatory theta activity also plays a role in movement production during cognitive control, action monitoring, *et al*.^72-76^

### Saliency/attention network impairments during healthy aging

Our findings on MS3 & MS4 provide evidence for saliency/attention network impairments from young to aging adults in support of PASA theory. The saliency network includes human brain composed of the anterior insular and dorsal anterior cingulate cortex. According to PASA theory, a neural shift from posterior to anterior is commonly detected. The modulations in alpha and beta oscillations at pre-central and post-central cortical sites have been linked to execution or voluntary activities. Alpha oscillations in parieto-occipital areas are that alpha activity, such as age-related reduction in alpha amplitude, purportedly associated with weakening of inhibitory abilities.^77-80^ Both alpha and beta power attenuation were reported to commence before voluntary movements in the fronto-medial and central areas.^81,82^ Furthermore, it was reported that elder adults exhibit more beta power during rest compared to young adults.^83^ Also, increased attenuation and recruitment of cortical areas occurred during self-paced movements in elders.^84^ In our study, the increased alpha occurrence/coverage and beta coverage in MS3 may be related to voluntary and execution declines across lifespan, moving anteriorly across lifespan. The greater attenuation of alpha/beta oscillations in elderly has been associated with enhanced involvement of neural resources for energy-consuming during executive responses.^85-87^ Our findings in MS3&MS4 may also indicate that low efficiency in neural activities appears with the increase of age.

### Alpha dysfunction in cognitive decline across lifespan

Three parameters (duration, occurrence, coverage) in machine-learning based microstate patterns provide information with temporal and spatial resolution. Consistent with our hypotheses, microstate patterns indicated age-related whole-brain dynamic neural activities impairments with dominant alpha responses, accompanying with related frequency variations. Both topographic and temporal dynamic microstate changes are useful to explore and predict aging in humans. For example, as previously suggested, decreased alpha power and peak frequency amplitude on scalp level was typically found in occipital-parietal areas with the increased age.^12,88^ Alpha power was linked to a variety of cognitive decline, such as attention, inhibition and memory retrieval. However, in this study, the decreased alpha duration and increased alpha occurrence provide insights into the temporal modulations at left-right topographic orientation.

## Strength and limitations

To sum up, there was several main findings according to this microstate analysis in the Cam-CAN cohort: 1) We found the dominant age-related alpha band microstate effects, especially an increased alpha occurrence in the whole brain with healthy aging. 2) The MS1&MS2 intra-subject variability explained that individualized microstate patterns at different age groups involved a higher-order sensory system in elder adults. 3) The microstate effects in MS3&MS4 implicate the dysfunction with healthy aging in saliency/attention network, moving anteriorly in elder adults. These findings provide insightful evidences for the reduced neural efficiency across lifespan.

The strength of this work is that we have identified novel biomarkers of microstate patterns and confirm that there are dominant alpha deficiencies with multi-frequency recession in the progression of aging. Furthermore, the Cam-CAN cohort provides a wide range of age groups to examine the progress of aging. The findings based on the gender-balanced cohort could convincingly reveal the oscillation changes with increased age. With high temporal and reasonable spatial resolution, both oscillations and topography across lifespan can be in-vivo detected. The limitation of the work is lack of tracking for the neural sources to uncover the relationships between microstate patterns and brain atrophy. In future work, it is essential to combine resting MEG, functional magnetic imaging resonance (MRI) and structural MRI to obtain multi-model aging biomarkers.

## Conclusions

In conclusion, we discovered oscillation changes across different age groups via microstate patterns, and the results suggested that aging involve alpha microstate impairments, accompanying with theta and beta changes. The identified novel biomarker may be helpful to predict aging in future.

## Supplementary Material

fcae150_Supplementary_Data

## Data Availability

Data are available in Github ‘https://github.com/huangyujing14/Brain-Communications’
