# Supplementary material for: Magnetoencephalography-derived oscillatory microstate patterns across lifespan: the Cambridge centre for ageing and neuroscience cohort
Source: Brain Commun. 2024 Apr 29;6(3):fcae150. doi: 10.1093/braincomms/fcae150 (PMC11091929; doi:10.1093/braincomms/fcae150)
Supplement: fcae150_Supplementary_Data [file fcae150_supplementary_data.zip › Supplementary Table 1.docx]

| **MS1** | **YA**  Supplementary Table 1. Microstate Pattern Parameter Changes with Healthy Aging    **N=67** | **EMA**  **N=141** | **LMA**  **N=150** | **YS**  **N=150** | **EA**  **N=116** | **One-way ANOVA**  **F (df1=4, df2=619)** | **Post-hoc**  **Tukey** | **Pearson’s r**  **(2-tailed)^b^** | **Beta** |
| --- | --- | --- | --- | --- | --- | --- | --- | --- | --- |
| Delta/coverage | 0.23(3E3) | 0.19(3E3) | 0.23(2E3) | 0.26(4E3) | 0.18(6E3) | F=54.83*** | YS>>>YA>>>EMA  YA=LMA>>>EA  EMA<<<LMA<<<YS  YS>>>EA(a) | n.s. | 0.87*** |
| Delta/duration | 0.41(3E3) | 0.39(2E3) | 0.41(2E3) | 0.43(5E3) | 0.39(1E2) | F=10.50*** | YS>>>YA>>>EMA  EMA<<<LMA  YS>>LMA(b)  YS>EA(b)(a) | n.s. | -0.31** |
| Delta/occurrence | 0.56(5E3) | 0.49(5E3) | 0.57(4E3) | 0.60(5E3) | 0.47(8E3) | F=88.36*** | YA>>>EMA  EA<<<YA<<<YS  EMA<<<LMA<<<YS  LMA>>>EA(a) | n.s. | -0.56*** |
| Theta/duration | 0.19(2E3) | 0.19(1E3) | 0.18(1E3) | 0.18(1E3) | 0.18(2E3) | F=4.33**^b^ | YA>LMA(b)  YA>>YS=EA(b)(a) | -0.17** | n.s. |
| Alpha/duration | 0.14(3E3) | 0.14(2E3) | 0.14(2E3) | 0.12(1E3) | 0.12(1E3) | F=15.06*** | YA=EMA>>>YS=EA  LMA>>EA(b)  LMA>>>YS(b)(a) | -0.29** | n.s. |
| Alpha/occurrence | 1.47(3E2) | 1.56(3E2) | 1.58(3E2) | 1.75(2E2) | 1.69(3E2) | F=10.14*** | YA=EMA<<<YS  LMA<<<YS(b)  YA<<<EA(b)  EMA<EA(b) | 0.22** | n.s. |
| Beta/occurrence | 4.79(3E2) | 4.72(2E2) | 4.61(2E2) | 4.58(2E2) | 4.60(2E2) | F=8.35*** | YA>>>YS  YA>>>LMA=EA(b)  EMA>LMA=EA(b)  EMA>>YS(b) | -0.20** | n.s. |
| Gamma/coverage | 0.24(5E3) | 0.24(2E3) | 0.23(4E3) | 0.22(4E3) | 0.21(5E3) | F=8.71*** | YA>>>YS=EA(b)  EMA>>>YS=EA(b)(a) | -0.23** | -2.44*** |
| Gamma/duration | 0.36(4E4) | 0.36(1E4) | 0.37(3E4) | 0.36(3E4) | 0.35(4E4) | F=2.95*^b^ | EMA<LMA(b)(a) | n.s. | 0.78*** |
| Gamma/occurrence | 6.70(8E2) | 6.69(4E2) | 6.18(6E2) | 5.98(6E2) | 5.94(8E2) | F=25.13*** | YA=EMA>>>LMA=YS=EA(a) | -0.34** | n.s. |
| **MS2** | **YA**  **N=67** | **EMA**  **N=141** | **LMA**  **N=150** | **YS**  **N=150** | **EA**  **N=116** | **One-way ANOVA**  **F (df1=4, df2=619)** | **Post-hoc**  **Tukey** | **Pearson’s r**  **(2-tailed)^b^** | **Beta** |
| Delta/coverage | 0.22(4E3) | 0.23(2E3) | 0.21(3E3) | 0.18(5E3) | 0.24(7E3) | F=21.71*** | YA>>>YS  EMA>>>LMA>>>YS  LMA<EA(b)  YS<<<EA(a) | n.s. | 0.57* |
| Delta/duration | 0.41(3E3) | 0.41(2E3) | 0.40(2E3) | 0.38(3E3) | 0.42(7E3) | F=14.79*** | YA=EMA=LMA=EA>>>YS(a) | n.s. | n.s. |
| Delta/occurrence | 0.53(6E3) | 0.56(4E3) | 0.52(6E3) | 0.47(1E2) | 0.55(9E3) | F=24.15*** | YA>>>YS  YA<<<EMA(b)  EMA>>>LMA  EMA>>>YS  LMA>>>YS(b)  YS<<<EA(a) | -0.12** | n.s. |
| Theta/duration | 0.18(1E3) | 0.18(1E3) | 0.18(1E3) | 0.18(1E3) | 0.18(1E3) | F=2.38*^b^ | YA>EA(b)(a) | -0.12** | n.s. |
| Alpha/coverage | 0.25(6E3) | 0.23(4E3) | 0.21(3E3) | 0.21(4E3) | 0.22(4E3) | F=11.87*** | YA>>>LMA=YS  YA>>EA(b)  EMA>>>LMA(b)  EMA>>>YS(b) | -0.16** | -0.51** |
| Alpha/duration | 0.15(4E3) | 0.15(2E3) | 0.13(2E3) | 0.12(1E3) | 0.12(1E3) | F=32.57*** | YA>>>LMA(b)  EMA>>>LMA  YA=EMA>>>YS=EA  LMA>YS(b)  LMA>>>EA(b)(a) | -0.40** | n.s. |
| Alpha/occurrence | 1.60(3E2) | 1.55(2E2) | 1.56(2E2) | 1.66(2E2) | 1.80(3E2) | F=13.55*** | YA<<<EA(b)  EMA<<<EA  EMA=LMA<YS(b)  LMA<<<EA  YS<<EA(b) | 0.23** | 0.64*** |
| Beta/duration | 0.05(6E4) | 0.05(3E4) | 0.05(4E4) | 0.05(5E4) | 0.05(5E4) | F=4.94***^b^ | YA<EA(b)  EMA<YS(b)  EMA<<<EA(b)(a) | 0.16** | n.s. |
| Beta/occurrence | 4.65(3E2) | 4.57(2E2) | 4.56(2E2) | 4.51(2E2) | 4.58(2E2) | F=2.51*^b^ | YA>YS(b)(a) | -0.08* | n.s. |
| Gamma/coverage | 0.22(4E3) | 0.22(2E3) | 0.25(2E3) | 0.24(3E3) | 0.25(5E3) | F=14.59*** | YA=EMA<<<LMA  YA=EMA<<<EA(b)  EMA<<<YS(b)  YA<<<YS(b)(a) | 0.22** | n/a |
| Gamma/occurrence | 6.00(7E2) | 6.08(4E2) | 6.82(5E2) | 6.61(5E2) | 6.66(5E2) | F=42.93*** | YA=EMA<<<LMA=YS=EA  LMA>YS(b) | 0.34** | -0.55* |
| **MS3** | **YA**  **N=67** | **EMA**  **N=141** | **LMA**  **N=150** | **YS**  **N=150** | **EA**  **N=116** | **One-way ANOVA**  **F (df1=4, df2=619)** | **Post-hoc**  **Tukey** | **Pearson’s r**  **(2-tailed)^b^** | **Beta** |
| Delta/coverage | 0.21(3E3) | 0.23(3E3) | 0.22(3E3) | 0.19(6E3) | 0.19(6E3) | F=14.12*** | EMA>>>YA  EMA>>LMA(b)  LMA>>>YS(b),  EMA>>>EA,EMA>>>YS  LMA>EA(b)(a) | -0.20** | 0.85** |
| Delta/occurrence | 0.53(5E3) | 0.57(4E3) | 0.55(5E3) | 0.47(6E3) | 0.48(1E2) | F=32.07*** | YS<<<YA<<<EMA  EMA>>LMA(b)  EMA>>>YS=EA  LMA>>>YS=EA  YA>EA(b)(a) | -0.32** | -0.55** |
| Theta/coverage | 0.23(3E3) | 0.24(3E3) | 0.23(3E3) | 0.22(5E3) | 0.24(4E3) | F=5.01***^b^ | EMA>>>YS(b)  EA>YS(b)(a) | n.s. | n.s. |
| Theta/duration | 0.20(2E3) | 0.20(1E3) | 0.19(1E3) | 0.19(2E3) | 0.19(2E3) | F=5.22***^b^ | EMA>LMA(b)  EMA>>YS=EA(b)(a) | -0.16** | n.s. |
| Theta/occurrence | 1.17(1E2) | 1.20(1E2) | 1.20(1E2) | 1.15(1E2) | 1.25(1E2) | F=10.01*** | YA=YS<<<EA  EMA>>YS(b)  EMA<EA(b)  LMA>YS(b)(a) | 0.07* | n.s. |
| Alpha/coverage | 0.16(6E3) | 0.18(5E3) | 0.19(6E3) | 0.21(6E3) | 0.21(7E3) | F=10.74*** | YA<<<YS=EA  YA<<<LMA(b)  EMA<<<YS(b)  EMA<<EA(b)(a) | 0.27** | n.s. |
| Alpha/duration | 0.13(2E3) | 0.13(2E3) | 0.13(1E3) | 0.13(1E3) | 0.12(1E3) | F=3.17*^b^ | EMA=LMA>EA(b) | n.s. | -0.26** |
| Alpha/occurrence | 1.23(4E2) | 1.36(3E2) | 1.45(3E2) | 1.64(3E2) | 1.68(4E2) | F=17.75*** | YA<LMA(b)  YA=EMA<<<YS=EA  LMA<<YS(b)  LMA<<<EA(b) | 0.33** | n.s. |
| Beta/coverage | 0.22(4E3) | 0.23(2E3) | 0.24(3E3) | 0.24(3E3) | 0.24(3E3) | F=7.45*** | YA<<<YS  YA<<<EA(b)  YA<<LMA(b)  EMA<YS(b) | 0.20** | n.s. |
| Beta/duration | 0.05(4E4) | 0.05(3E4) | 0.05(3E4) | 0.05(3E4) | 0.05(3E4) | F=14.43*** | YA<EMA(b)  YA<<<LMA=YS=EA  EMA<<<YS(b)  EMA<<EA(b) | 0.27** | n.s. |
| **MS4** | **YA**  **N=67** | **EMA**  **N=141** | **LMA**  **N=150** | **YS**  **N=150** | **EA**  **N=116** | **One-way ANOVA**  **F (df1=4, df2=619)** | **Post-hoc**  **Tukey** | **Pearson’s r**  **(2-tailed)^b^** | **Beta** |
| Delta/coverage | 0.33(5E3) | 0.33(5E3) | 0.32(7E3) | 0.35(7E3) | 0.37(1E2) | F=6.87*** | YA<EA(b)  EMA=LMA<<EA(b)  LMA<YS(b)(a) | 0.16** | n/a |
| Delta/duration | 0.49(5E3) | 0.49(5E3) | 0.49(7E3) | 0.52(9E3) | 0.57(2E2) | F=8.89*** | YA=EMA=LMA<<EA(b)  EMA<YS(b)(a) | 0.20** | n.s. |
| Delta/occurrence | 0.67(6E3) | 0.66(5E3) | 0.64(5E3) | 0.67(7E3) | 0.65(7E3) | F=3.89**^b^ | YA>LMA(b)  YS>LMA(b) | n.s. | 0.22** |
| Theta/coverage | 0.31(6E3) | 0.30(5E3) | 0.31(6E3) | 0.33(7E3) | 0.30(8E3) | F=2.63*^b^ | EMA<YS(b)(a) | n.s. | n/a |
| Theta/occurrence | 1.38(1E2) | 1.37(1E2) | 1.40(1E2) | 1.42(1E2) | 1.38(1E2) | F=2.76*^b^ | EMA<YS(b) | n.s. | n.s. |
| Alpha/occurrence | 1.76(3E2) | 1.84(2E2) | 1.86(2E2) | 1.99(2E2) | 1.95(3E2) | F=10.43*** | YA<<<YS  EMA<<<YS(b)  YA<<<EA(b)  EMA<EA(b)  LMA<<<YS(b) | 0.23** | n.s. |
| Beta/coverage | 0.28(4E3) | 0.28(3E3) | 0.27(3E3) | 0.26(3E3) | 0.26(3E3) | F=7.12*** | YA=EMA>YS(b)  YA>>>EA(b)  EMA>>>EA(b)  LMA>EA(b) | -0.21** | n/a |
| Beta/duration | 0.05(5E4) | 0.05(4E4) | 0.05(4E4) | 0.05(3E4) | 0.05(4E4) | F=5.00***^b^ | YA=EMA>>EA(b)  LMA>EA(b) | -0.18** | n.s. |
| Beta/occurrence | 5.01(5E2) | 4.99(3E2) | 4.90(3E2) | 4.83(4E2) | 4.77(4E2) | F=4.89***^b^ | YA>EA(b)  EMA>YS(b)  EMA>>EA(b) | -0.17** | n.s. |
| Gamma/coverage | 0.27(6E3) | 0.27(2E3) | 0.26(3E3) | 0.27(3E3) | 0.28(4E3) | F=5.28***^b^ | LMA<<EMA=YS(b)  LMA<<<EA(b)(a) | n.s. | -2.16** |
| Gamma/duration | 0.03(5E4) | 0.03(2E4) | 0.03(3E4) | 0.03(2E4) | 0.03(3E4) | F=3.70**^b^ | LMA<YS(b)  LMA<<EA(b) | n.s. | n.s. |
| Gamma/occurrence | 6.91(7E2) | 7.08(3E2) | 6.85(4E2) | 7.03(5E2) | 7.09(5E2) | F=4.59***^b^ | LMA<YS(b)  LMA<<EA=EMA(b) | n.s. | n.s. |

Notes:The symbols of ’*’, ** and *** indicate that significance level at the 0.05, 0.01, and 0.001 respectively; the symbols of ‘>’,’>>’, and ‘>>>’ mean that significance level at the 0.05, 0.01, 0.001 respectively; the symbols of ‘<’, ‘<<’ and ‘<<<’ mean that significance level at the 0.05 0.01, 0.001 respectively; ‘n.s.’ means there was no significance. The values of ‘mean(S.E.)’ was provided for each age group (YA: young adults; EMA: early middle age; LMA: late middle age; YS: young seniors; EA: elder adults). The letter ‘(a)’ means Tamhane’s T2 was used due to heterogeneity of variances. Pearson’s correlations between age and microstates parameters were calculated. The superscript letter ‘b’means that the statistics didn’t pass the Bonferroni correction. Beta of linear regression analyses indicated the standardized coefficients with age as dependent variable and all microstate parameters as independent variables.
